# Impact in Clinical Practice of the European Medicines Agency Health Alert About the Restriction of the Use of JAK Inhibitors

**DOI:** 10.3390/ph18010022

**Published:** 2024-12-27

**Authors:** Elisabet Castañeda-Estévez, Cristina Vergara-Dangond, Martina Steiner, Maria Beatriz Paredes-Romero, Ana Esteban-Vázquez, Tatiana Cobo-Ibañez, Laura Trives-Folguera, Maria Liz Romero-Bogado, Isabel De La Cámara-Fernández, Patricia Richi-Alberti, Ana Acosta-Alfaro, Iría De la Osa-Subtil, Santiago Muñoz-Fernández

**Affiliations:** 1 Department of Rheumatology, Hospital Universitario Infanta Sofía, FIIB HUIS HHEN, Universidad Europea, 28702 Madrid, Spain; elisabet2993@gmail.com (E.C.-E.); martina.steiner@salud.madrid.org (M.S.); mbeatriz.paredes@salud.madrid.org (M.B.P.-R.); ana.esteban@salud.madrid.org (A.E.-V.); mtatiana.cobo@salud.madrid.org (T.C.-I.); laura.trives@salud.madrid.org (L.T.-F.); mromerob@salud.madrid.org (M.L.R.-B.); isabeldela.camara@salud.madrid.org (I.D.L.C.-F.); patricia.richi@salud.madrid.org (P.R.-A.); anavaleria.acosta@salud.madrid.org (A.A.-A.); santiago.munoz@salud.madrid.org (S.M.-F.); 2 Department of Medicine, Faculty of Biomedical and Health Sciences, European University of Madrid, 28670 Madrid, Spain; iria.delaosa@universidadeuropea.es

**Keywords:** JAK inhibitors, cardiovascular risk, health alert

## Abstract

**Background/Objectives:** Janus kinase inhibitors (JAKi) have revolutionized the treatment of various inflammatory and immune disorders. Concerns about the potential increased risk of major adverse cardiovascular events (MACEs) associated with JAKi use led to a European Medicines Agency (EMA) health alert recommending restricting the use of JAKi in high-risk populations. **Methods:** This study aims to determine the proportion of patients who developed any cardiovascular, ischemic, neoplastic, or thrombotic adverse event in a cohort of patients receiving, or who have received, JAKi treatment between January 2017 and September 2023. In addition, we studied the impact of the alert in the clinical practice of our Rheumatology department. **Results:** In this retrospective, observational study, 101 patients were analysed, predominantly women (72.3%), with a mean age of 57.6 years. The most common diagnoses were rheumatoid arthritis (60.4%) and psoriatic arthritis (18.8%). The most frequent adverse events were infections (5.9%) and neoplasms (2.9%). Deep vein thrombosis and haemorrhagic stroke each occurred in 1% of patients, with no cases of ischemic stroke or ischemic heart disease. When the health alert was published, 75 patients were on treatment with JAKi, and 33 met the alert criteria. Of these, 12.1% changed treatment due to the alert, 9% had no other therapeutic options, 57.5% were in clinical remission or had low disease activity, and 9% maintained treatment at their own request. **Conclusions:** These findings suggest that, in this patient cohort, JAKi use did not result in new MACE cases. The alert concerning the use of JAKi has had a limited impact in our clinical practice.

## 1. Introduction

The development of immunosuppressive therapies, such as Janus kinase inhibitors (JAKi), has marked a significant change in the treatment of various inflammatory and immune disorders. JAKi inhibit intracellular signalling pathways controlled by various cytokines, thus preventing the aberrant immune responses involved in diseases such as rheumatoid arthritis, psoriatic arthritis, spondyloarthritis, and systemic lupus erythematosus, among others [1].

The four Janus kinase proteins and the Signal Transducers and Activators of Transcription (STAT) signalling pathway regulate the cellular responses to cytokines, interferons, and growth factors by influencing intracellular signals to the nucleus and activating gene expression. JAKs are members of the intracellular nonreceptor protein tyrosine kinase family, which includes four JAKs (JAK1–3 and TYK2). Upon binding of the ligand to the surface receptor, the dimerization of the JAK-associated receptor induces the activation of JAK kinase, which, in turn, recruits and phosphorylates cytosolic STAT proteins and leads to the nuclear translocation of STAT, acting as a transcription factor. JAK-mediated phosphorylation of STAT results in the formation of homo- or heterodimers, and activated STAT is then translocated into the nucleus to regulate genes containing response sequences. Numerous pro-inflammatory cytokines contribute to JAK/STAT pathway activation. The binding of different cytokines to JAK-associated receptor subunits results in the stimulation of specific downstream intracellular signals that play critical roles in the pathogenesis of immune diseases [1].

To date, four oral JAKi have been approved for use in rheumatological immune-mediated diseases (IMIDs): upadacitinib and filgotinib, which are selective for JAK-1; baricitinib, which is selective for JAK-1 and JAK-2; and tofacitinib, which is a pan-JAK inhibitor (JAK-1, JAK-3, and, to a lesser extent, JAK-2) [2].

It has recently been proposed that patients treated with tofacitinib may have an increased risk of developing major adverse cardiovascular events (MACEs), such as ischemic or thrombotic events, and malignancies [3]. This proposal follows the observation of an increased incidence of these events in the preliminary results of the ORAL Surveillance safety trial. This trial included a population of 4362 rheumatoid arthritis (RA) patients over 50 years of age with at least one cardiovascular risk factor, defined in the protocol as current cigarette smokers, high blood pressure, and high-density lipoprotein (HDL). The trial compared the risks of MACEs and malignancies between tofacitinib users and patients using tumour necrosis factor inhibitors (TNFi). Slight increases in the MACE and malignancy ratios of 1.33 (0.91–1.34) and 1.48 (1.04–2.09), respectively, were found in patients treated with tofacitinib [4].

For this reason, in October 2022, the European Medicines Agency (EMA) published a health alert extended to all JAKi drugs for possible class effects, even though these data have not been demonstrated in other clinical trials to date4. In this alert, the EMA recommended avoiding the use of these drugs in patients who are aged 65 years or older, have an increased cardiovascular risk (a history of myocardial infarction or stroke), are smokers or former long-term smokers, and have an elevated risk of cancer, allowing their use only when there is no other alternative treatment [5].

Our objective was to study the safety profile of JAKi and to determine the percentage of development of MACEs, such as stroke or ischemic heart disease, as well as the development of neoplasms in the different subgroups of patients who receive or have received a JAKi in our Rheumatology department. In addition, we studied the impact of the EMA alert on our clinical practice in patients treated with JAKi.

## 2. Patients and Methods

### 2.1. Study Design and Population

For our study, we designed a retrospective, observational, descriptive, and longitudinal study.

Patients from the Rheumatology department of the Hospital Universitario Infanta Sofía (HUIS) in Madrid, who received or have received treatment with JAKi between 1 January 2017, and 30 September 2023, were included. Patients with inflammatory diseases receiving other drug treatments were excluded. Demographic variables such as age and sex, and clinical variables such as medical history (hypertension, diabetes, dyslipidaemia, neoplasms, stroke, smoking, and alcohol consumption), rheumatologic diagnosis, time of disease diagnosis, previous use of immunosuppressive drugs, duration of JAKi use, adverse events, and reasons for JAKi change were collected from medical records.

After the date of publication of the EMA alert, we carefully evaluated all patients treated with JAKi. All patients who met the alert criteria were informed and were offered another therapeutic option, if possible. Patients in which other therapeutic options were used and failed and those who chose to continue with JAKi due to good response to the therapy were informed about the EMA alert. In these cases, the final decision to continue the treatment was made by the patient.

### 2.2. Statistical Analysis

To achieve the objectives of this study, a descriptive analysis was conducted, using absolute frequencies (n) and relative frequencies (%) for qualitative variables, and mean and standard deviation (SD) for quantitative variables. The normal distribution of quantitative variables was assessed using the Kolmogorov–Smirnov test and the Q-Q plot. If a normal distribution was not maintained, the median, interquartile range (IQR), and percentiles Q1, Q2, and Q3 were obtained. A statistical analysis was performed using Jamovi software (version 2.3.21).

## 3. Results

A total of 101 patients were analysed. The majority were women (72.3%), with a mean age of 57.6 years (SD 12.57). Comorbidities are shown in Table 1, the most frequent of which were hypertension, diabetes mellitus, and dyslipidaemia. Furthermore, 15.8% were active smokers and 13.9% were ex-smokers. In total, 5.9% of patients developed a neoplasm during JAKi treatment (50% were colorectal, 33.3% dermatologic, and 16.7% hematologic).

Regarding diagnoses, 60.4% had RA, 18.8% had psoriatic arthritis (PsA), 5.9% had axial spondyloarthritis (ASpa), 3% had peripheral spondyloarthritis, 5.9% had seronegative arthritis, 3% had spondyloarthritis associated with inflammatory bowel disease, 2% had juvenile idiopathic arthritis, and 1% had systemic lupus erythematosus.

**Figure 1:**
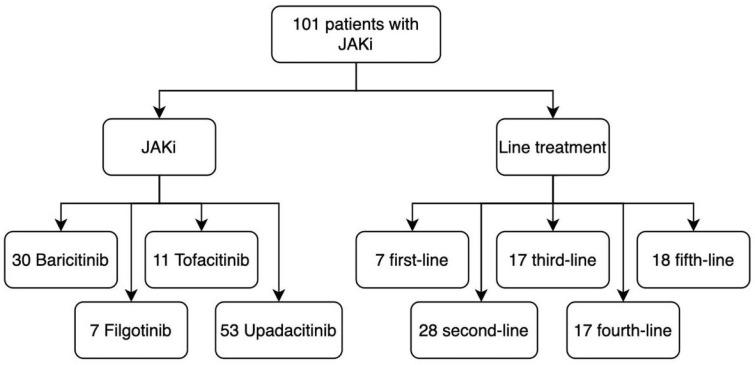
The distribution of Janus kinase inhibitor (JAKi) use and the lines of treatment for which they were selected in a population of 101 patients. Upadacitinib (53 patients) was the most commonly used treatment. In addition, the majority of patients were in the second-line of treatment (28 patients).

Of the total of patients, 7.2% started treatment with a JAKi as first-line treatment, 27.7% as a second-line treatment, 29.7% as a third-line treatment, and 16.8% and 17.8% as a fourth- or fifth-line treatment, respectively. Additionally, 29.7% were on treatment with baricitinib, 6.9% with filgotinib, 10.8% with tofacitinib, and 52.5% with upadacitinib (Figure 1). The median duration of all the drugs used was 26 months (minimum of 0 months and maximum of 81 months), with an interquartile range of 16 months.

The most frequent adverse events observed were infections that forced discontinuation of the drug in 5.9% (including varicella zoster, repeated respiratory infections, and septic arthritis). Neoplasms were observed in 2.97% of patients, including colorectal cancer, prostate cancer, and Kaposi’s sarcoma. Deep vein thrombosis (DVT) of the lower limbs and haemorrhagic stroke in an anticoagulated patient appeared in 1%, respectively. Other side effects, such as digestive intolerance, headache, weight gain, cough, or menstrual cycle alterations, appeared in 11.9%. No patient developed an ischemic stroke or an ischemic heart disease during JAKi use (Table 2).

We hypothesized about the influence of sex in the appearance of adverse events. We did not find any significant difference between sexes.

**Figure 2:**
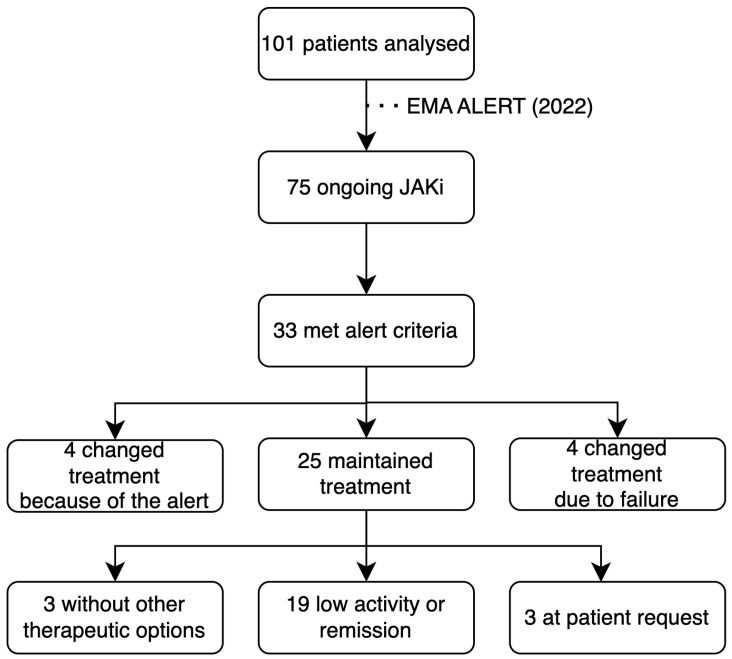
The response to the health alert in the Rheumatology department. At the time of the alert, there were 75 patients receiving treatment with JAK inhibitors (JAKi). Of these, 33 met the alert criteria.

In 2022, when the health alert was published by the EMA, 75 patients were on treatment with JAKi, of whom 33 met the alert criteria. Among these patients who met the alert criteria, 4 (12.1%) changed treatment because of the alert, 4 (12.1%) changed due to treatment failure, 3 (9.1%) maintained treatment because there were no other therapeutic options, 19 (57.6%) maintained the JAKi because of being in clinical remission or having low disease activity, and 3 (9.1%) maintained treatment at their own request. All patients in whom JAKi were maintained had agreed to continue with the treatment after having been informed about the EMA alert (Figure 2).

## 4. Discussion

JAKi are a family of phosphotransferases originally identified in the early 1990s; they possess tandem kinase domains, making them plausible targets for the signalling of numerous cytokines. Many of these cytokines play important roles in the pathogenesis of immune-mediated diseases, thus providing the rationale for JAK inhibition as a therapeutic approach [6].

Tofacitinib was the pioneer JAKi approved for rheumatological and gastroenterological conditions, with the first approval received in 2012 for RA [7]. Since then, other JAKi have appeared for immune-mediated diseases with higher JAK selectivity, such as baricitinib (being selective for JAK-1 and JAK-2), as well as upadacitinib and filgotinib (known as second-generation JAKi, which are selective for JAK-1). New molecules with greater selectivity for single JAKs have been approved, with the goal of improving safety profiles while maintaining efficacy. These drugs appeared to be safe in clinical trials. In the near future, more JAKi will be available for the treatment of various immune-mediated diseases.

The ORAL Surveillance safety trial studied the risk of developing a MACE, such as ischemic or thrombotic events, and cancer in RA patients treated with tofacitinib. This trial compared patients treated with tofacitinib versus two TNF inhibitors (adalimumab and etanercept) in a large population of 4362 RA patients over 50 years of age with at least one cardiovascular risk factor, such as current cigarette smoker, high blood pressure, or high-density lipoprotein (HDL). The study showed increased risks of a MACE (1.33; 0.91–1.34) and malignancy (1.48; 1.04–2.09) in patients treated with tofacitinib [4].

Considering this result, the EMA published a health alert in 2022, extended to all JAKi drugs for possible class effects. In this alert, the EMA recommended avoiding the use of these drugs in patients aged 65 years or older, with increased cardiovascular risk, smokers or former long-term smokers, and with elevated risk of cancer, allowing their use only when there is no other alternative treatment [5]. The increase in risk has not been yet confirmed with other JAKi than tofacitinib.

Long-term extension studies with tofacitinib, baricitinib, upadacitinib, and filgotinib in RA, as well as the post-hoc analysis of tofacitinib and upadacitinib in patients with RA, PsA, ASpa, or atopic dermatitis did not show any increased risk of MACEs or DVT. Interestingly, the most selective second-generation JAKi do not seem to be associated with any increased incidence of MACEs, DVT, or malignancies. Notably, MACEs and DVT were reported in patients with rheumatological conditions, suggesting a role of the underlying disease [6].

In 2023, a study addressing the safety profile of upadacitinib over 15,000 patient-years in RA, PsA, ASpa, or atopic dermatitis was published, describing an increased risk of herpes zoster and elevated CPK levels, consistent with the overall safety profiles of JAK inhibitors; otherwise, no new safety risks were identified when compared to previous reports [8].

In April 2024, the BIOBADASER registry comparing the safety of JAKi with that of tumour necrosis factor inhibitors (TNFi) in 6826 patients was published, which revealed significant differences between patients treated with JAKi and TNFi in a real-life setting, associated with a small increase in the frequency of adverse events—in particular, infections, herpes zoster, and gastrointestinal events—but these were mild and without increased mortality. Concerning MACEs, they did not describe an increased incidence in patients on JAKi treatment [9].

A study based on real-world evidence regarding the safety profile of upadacitinib in RA, PsA, and ASpa up to 5 years, including a total of 1789 patients, was also published in 2024. In the PsA group, higher rates of serious infection, herpes zoster, lymphopenia, and non-melanoma skin cancer were reported with upadacitinib versus adalimumab; meanwhile, MACEs and venous thromboembolic events were comparable between upadacitinib and adalimumab in PsA, and they were similar in all diseases [10].

A Japanese group also conducted a safety study on the use of baricitinib in patients with rheumatoid arthritis in clinical practice in 2024, in which 4720 patients were analysed. They determined that there is an increased risk of herpes zoster infections and other serious infections that must be taken into account with its use, without demonstrating an increase in the incidence of MACEs in these patients [11].

Whether JAKi use is associated with an increased risk of cardiovascular events is yet to be defined. It seems that carrying cardiovascular risk factors or previous atherosclerotic cardiovascular disease along with smoking and older age leads to a greater risk in patients, as well as having an underlying inflammatory disease that is not well controlled.

Following the health alert regarding JAKi, the restrictions on their use recommended by the EMA are already reflected in the current EULAR recommendations for managing rheumatoid arthritis, although data on the increased risk of MACEs have only been collected from a single large study so far [4,12].

To the best of our knowledge, this is the first study that aims to evaluate the impact of the EMA alert on a population of patients treated with JAKi. In our cohort, only 4 (12.1%) of the patients that fulfilled the alert criteria were changed to another therapeutic option because of the alert, while the majority maintained JAKi treatment. This option was chosen by the patients as they were characterized by low disease activity or remission, there were no other therapeutic options, or due to their preference. The patients, who were extensively informed about the EMA alert, made the final decision. We found no other published studies in the existing literature that have specifically addressed the impact of the EMA alert in the same context, limiting direct comparisons with our findings.

The last published morbidity registry of cardiovascular disease in Spain (2006) reported incidence rates of ischemic heart disease ranging from 135 to 210 new cases per 100,000 men and from 29 to 61 per 100,000 in women aged 25 to 74 years. In addition, the incidence of cerebrovascular disease for both sexes ranges between 120 and 350 cases per 100,000 inhabitants per year, being lower in women (169/100,000) than in men (183–364/100,000), and multiplying by 10 in the population over 70 years of age [13].

In our study population, we did not detect the development of MACEs, although our patient population did not meet the expected cases for these pathologies. However, there were cases of neoplasms developing during treatment with JAKi, which made discontinuation of treatment necessary.

The possible influence of sex on the appearance of MACEs and cancer was considered, but we did not find any significant differences, possibly due to the small number of cases between the groups.

Furthermore, data from a German observational data analysis (RABBIT) describing the risk of developing MACEs in a population of more than 8000 rheumatoid arthritis patients on biologic disease-modifying antirheumatic drug (bDMARD) treatment, JAKi, and conventional synthetic disease-modifying antirheumatic drugs (csDMARDs) have recently been published (in October 2023). Adjusted risks comparing JAKi, bDMARDs, and csDMARDs with TNFi were 0.89 (0.52 to 1.52), 0.76 (0.45 to 1.27), and 1.36 (0.85 to 2.19) overall, and 0.74 (0.41 to 1.31), 0.75 (0.45 to 1.27), and 1.21 (0.74 to 1.98) in cardiovascular risk patients. The risk was not increased in patients ≥ 65 years, patients with a cardiovascular history, or smokers and not when using a csDMARD as a reference instead of a TNFi. The risk values for baricitinib, tofacitinib, and upadacitinib were 0.49 (0.25 to 0.85), 0.98 (0.58 to 1.55), and 0.53 (0.15 to 1.36), respectively. The authors concluded that treatment with JAKi was not associated with an increased risk of MACEs, even in subgroups with higher-risk profiles [3]. This finding disagrees with the results of the ORAL Surveillance study [4], and instead, aligns with our results.

It is important to acknowledge that our study has limitations, mostly due to the small sample size. To draw more robust conclusions, it would be beneficial to include larger patient populations with similar characteristics in future research efforts. Expanding the study to include a broader cohort could provide more comprehensive data on the safety profile of JAKi and the impact of health alerts on clinical practice.

## 5. Conclusions

Despite the inferred risk associated with the use of JAKi, no new cardiovascular events or ischemic strokes were recorded in our group of patients in current clinical practice. This finding is consistent with the latest data published in the RABBIT study. In addition, a significant majority of patients who met the EMA health alert criteria continued treatment with JAKi, suggesting that the alert has had a relatively modest impact on clinical decisions. The decision to continue JAKi was primarily influenced by factors such as disease remission or low disease activity, limited alternative treatment options, and patient preference, reflecting a personalized approach to treatment continuation.

The limited occurrence of MACEs, despite the potential class effect concerns raised by the EMA alert, also underscores the need for further studies in larger cohorts to confirm the real-world safety profile of JAKi. This will help in understanding whether the risks observed in larger clinical trials are truly reflective of the broader patient population receiving JAKi, or whether other factors, such as comorbidities or lifestyle, play a more significant role in the development of adverse events.

It is worth noting that, even though our study sample was predominantly female, the small size of the cohort limited our ability to detect sex-based differences in adverse event risk with statistical significance. Moreover, the follow-up period in our study was relatively short, which may have prevented the detection of long-term adverse events associated with JAKi. Therefore, it would be desirable to increase the number of patients and extend the follow-up period in order to draw more robust conclusions.

The conclusions of this study should be taken with caution due to the small number of patients considered.

## Figures and Tables

**Table 1:** Demographic characteristics.

Characteristic	Total Group	Health Alert Subgroup (Yes)	Health Alert Subgroup (No)
n	101	33	42
**Mean age (years)**	57.6	64.0	53.5
**Female (%)**	72.3	69.7	76.2
**Hypertension (%)**	24.8	27.3	11.9
**Diabetes (%)**	13.9	9.1	14.3
**Dyslipidemia (%)**	20.8	33.3	9.5
**Smoker (%)**	15.8	33.3	0
**Ex-smoker (%)**	13.9	30.3	4.7
**Heart failure (%)**	4	9.1	0
**Ischemic heart disease (%)**	5	6.1	0
**Stroke (%)**	4	9.1	0
**Neoplasms (%)**	5.9	12.1	0

**Table 2:** Adverse events.

Adverse Events	Upadacitinib (53)	Tofacitinib (11)	Baricitinib (30)	Filgotinib (7)
**Infections (%)**	9.4	0	6.7	0
**Neoplasms (%)**	1.9	9.1	3.3	0
**Ischemic heart disease (%)**	0	0	0	0
**Ischemic stroke (%)**	0	0	0	0
**Hemorrhagic stroke (%)**	1.9	0	0	0
**DVT (%)**	0	0	3.3	0
**Others (%)**	9.4	0	10	42.8

## Data Availability

Data are contained within the article.
